# Chronic escitalopram treatment attenuated the accelerated rapid eye movement sleep transitions after selective rapid eye movement sleep deprivation: a model-based analysis using Markov chains

**DOI:** 10.1186/s12868-014-0120-8

**Published:** 2014-11-19

**Authors:** Diána Kostyalik, Szilvia Vas, Zita Kátai, Tamás Kitka, István Gyertyán, Gyorgy Bagdy, László Tóthfalusi

**Affiliations:** Department of Pharmacodynamics, Semmelweis University, Budapest, Hungary; MTA-SE, Neuropsychopharmacology and Neurochemistry Research Group, Budapest, Hungary; Department of Neurophysiology, Gedeon Richter Plc., Gyömrői út 19-21, Budapest, Hungary; Department of Behavioural Pharmacology, Gedeon Richter Plc., Gyömrői út 19-21, Budapest, Hungary

**Keywords:** REM sleep deprivation, EEG, Serotonin, Markov chain model, Stage transition, Animal model, Depression, Anxiety, Theta, REM sleep fragmentation

## Abstract

**Background:**

Shortened rapid eye movement (REM) sleep latency and increased REM sleep amount are presumed biological markers of depression. These sleep alterations are also observable in several animal models of depression as well as during the rebound sleep after selective REM sleep deprivation (RD). Furthermore, REM sleep fragmentation is typically associated with stress procedures and anxiety. The selective serotonin reuptake inhibitor (SSRI) antidepressants reduce REM sleep time and increase REM latency after acute dosing in normal condition and even during REM rebound following RD. However, their therapeutic outcome evolves only after weeks of treatment, and the effects of chronic treatment in REM-deprived animals have not been studied yet.

**Results:**

Chronic escitalopram- (10 mg/kg/day, osmotic minipump for 24 days) or vehicle-treated rats were subjected to a 3-day-long RD on day 21 using the flower pot procedure or kept in home cage. On day 24, fronto-parietal electroencephalogram, electromyogram and motility were recorded in the first 2 h of the passive phase. The observed sleep patterns were characterized applying standard sleep metrics, by modelling the transitions between sleep phases using Markov chains and by spectral analysis.

Based on Markov chain analysis, chronic escitalopram treatment attenuated the REM sleep fragmentation [accelerated transition rates between REM and non-REM (NREM) stages, decreased REM sleep residence time between two transitions] during the rebound sleep. Additionally, the antidepressant avoided the frequent awakenings during the first 30 min of recovery period. The spectral analysis showed that the SSRI prevented the RD-caused elevation in theta (5–9 Hz) power during slow-wave sleep. Conversely, based on the aggregate sleep metrics, escitalopram had only moderate effects and it did not significantly attenuate the REM rebound after RD.

**Conclusion:**

In conclusion, chronic SSRI treatment is capable of reducing several effects on sleep which might be the consequence of the sub-chronic stress caused by the flower pot method. These data might support the antidepressant activity of SSRIs, and may allude that investigating the rebound period following the flower pot protocol could be useful to detect antidepressant drug response. Markov analysis is a suitable method to study the sleep pattern.

## Background

Disturbed sleep pattern might be involved in the onset and course of depression [1]. Depressed patients often suffer from insomnia [2], and the polysomnographic recordings showed reduced interval between the sleep onset and the occurrence of the first rapid eye movement (REM) sleep episode (i.e. REM latency) [3], enhanced REM sleep amount, prolongation of the first REM episode, reduction of slow wave sleep (SWS), disturbed sleep continuity and greater number of stage shifts (see [4–6] for review). REM sleep disregulation is specific for depression, and it has been suggested to constitute a vulnerability marker to relapse or reoccurrence [1]. Similarly to depression, increased REM sleep pressure has also been observed in several animal models of depression applying acute or sub-chronic stress exposure [7–9], as well as during the recovery sleep after REM sleep deprivation (RD) [10–13] using the single platform on water (flower pot) protocol [14]. Besides RD, the flower pot method definitely causes a very stressful pathological condition due to isolation, immobilization, falling into the water, soaking, etc. [15]. In addition, total sleep suppression in different fashion produced anxiety in healthy humans [16–18] and anxiety-like behaviour in normal rodents [15,19,20]. Although sleep deprivation (SD) protocols may induce antidepressant effect in depressed patients and a reversal of depressive-like behaviours in animal models of depression [21–24]. Unfortunately, the mood improvement is only transient; relapses usually occur with the first episode of sleep, which is associated with high REM sleep pressure [25]. Antidepressant medication prevented the rapid relapse into depression after SD, and increased the efficacy of repeated SD [23,26,27]. Sleep deprivation is not a well-established model of depression yet, although the platform method can provoke anhedonic behaviour [28] and many studies have shown that it alters important pathways related to stress (see in review: [29]); additionally, chronic sleep loss may lead to stress-related mental disorders [30,31].

It is widely accepted that stress can cause sleep and REM sleep fragmentation [32–34]; however, solely a few studies have investigated the sleep phase transitions quantitatively. Although typical sleep metrics such as the number or the average duration of REM sleep episodes are less than perfect tools for this purpose. One of the reasons is that metrics are trying to grab only one-one feature of the observed hypnograms and compare treatment groups’ means simultaneously. But simultaneous testing inflates the alpha error and also can yield results which are very difficult to interpret in a coherent way. Furthermore, when the experimental groups are compared using metrics the degrees of freedom depend on the number of animals in each group and they are independent from the number of observations and from the length of the observed period. In statistical sense, metrics reduce a multivariate problem to a single variate one. This is a possible approach but generally not an efficient one in statistical sense. A possible solution to these problems is shifting from the descriptive statistical method to methods which connect more closely to the underlying biological processes. The idea that hypnogram, an apparently random sequence of WAKE-NREM-REM episodes can be described with the help of Markov chains goes back to the eighties [35] but the computational constraints prevented wide spread use of this method. Markov-modelling is essentially a regression-like procedure with the distinctive feature that outcome variables are modelled not as function-independent variables like time, but as a function of the previous observations. The goal of the regression procedure is to determine parameters which predict that the animal will be in a given stage, for example in REM stage, provided that the animal was in NREM. Of course, additional independent variables can be included into the model. The method is computationally intensive, because the model is fitted to all observations in a single run. But exactly because of that, all constraints between the parameters are naturally handled and the statistical assessments are based on all observations. The result of Markov analysis is a single coherent model which is optimal in statistical sense, because the model parameters are obtained by maximizing the likelihood. However, contrary to these appealing features, application of Markov models is still limited in the sleep research community [36–40]. We think that potential reasons of this backlog are the perceived hardware resource requirements and the lack of appropriate Markov module in the standard statistical program packages. But as we demonstrate, these hardware and software limitations are rapidly diminishing.

In this study, we explored how the chronically-administered (24 days) SSRI, escitalopram affects sleep and stage transitions during the first 2 h of rebound sleep after a 3-day-long RD using the flower pot method. To elucidate the transition processes between the vigilance stages, we modelled the observed sleep stages with time-continuous Markov chains. Unlike previous reports, we used freely available software tools for Markovian sleep analysis. Some practical hints about how to use these tools for hypnogram analysis are given in Methods section. Thus, the aim of this paper is twofold. Firstly, to provide further information on the mechanism of action of escitalopram, and secondly to demonstrate the applicability of hypnogram-modelling in answering a concrete research question.

## Methods

### Animal maintenance

All animal experiments and housing conditions were carried out in accordance with the European Community Council Directive of 24 November 1986 (86/609/EEC) and the National Institutes of Health “Principles of Laboratory Animal Care” (NIH Publications No. 85–23, revised 1985), as well as specific national laws (the Hungarian Governmental Regulation on animal studies, 31 December Psychopharmacology 1998 Act). All experiments were approved by the National Scientific Ethical Committee on Animal Experimentation and permitted by the government (Food Chain Safety and Animal Health Directorate of the Central Agricultural Office, Permit no. 22.1/1375/7/2010). All surgery was performed under anesthesia, and all efforts were made to minimize suffering. Male Wistar rats (n = 27) were purchased from Animal Facility (Semmelweis University, Budapest, Hungary). Rats, weighing 250 to 280 g at the surgery, were kept under controlled environmental conditions (temperature at 21 ± 1°C, and a 12 h light–dark cycle starting at 10:00 A.M.). Food and water were available *ad libitum* during the whole experiment.

### Surgery

Animals were equipped with electroencephalographic (EEG) and electromyography (EMG) electrodes as described earlier [41]. Briefly, stainless steel screw electrodes were implanted epidurally over the left frontal cortex (L: 2.0 mm and A: 2 mm to bregma) and left parietal cortex (L: 2.0 mm and A: 2.0 mm to lambda) for fronto-parietal EEG recordings. The ground electrode was placed over the cerebellum. In addition, EMG electrodes (stainless steel spring electrodes embedded in silicon rubber, Plastics One Inc., Roanoke, VA) were placed in the muscles of the neck. Surgery was performed under halothane (2%) anaesthesia (Fluotec 3) using a Kopf stereotaxic instrument.

For the chronic SSRI treatment, osmotic minipumps were implanted subcutaneously under the skin of the back of each animal, slightly posterior to the scapulae, through a 2 cm incision under halothane anaesthesia.

### Drug administration

Escitalopram-oxalate solution (10 mg/kg/day, kindly provided by Gedeon Richter Plc., dissolved in solution of 0.3 N HCl in distilled water) or its solvent was administered via ALZET osmotic minipumps (2ML4, ALZET, 2.5 μl per hour, 28 days DURECT Corporation, USA) continuously throughout the study. The dose of escitalopram was calculated based on chronic rodent studies demonstrating the effect of escitalopram on sleep, behaviour and extracellular 5-HT concentration [42–45].

### Habituation

To habituate the animals to the recording conditions, after a 7-day-long recovery period each rat was kept in a square, glass chamber (recording cage) separately and was attached to the polygraph by a flexible recording cable and an electric swivel fixed above the cages, permitting free movement. Habituation to the recording conditions lasted for 7 days and rats were connected to the cables during the whole period.

### REM sleep deprivation (RD) procedure

After the habituation period, rats were detached from the electric cable and the 72 h-long RD procedure was performed as described earlier [11]. Briefly, the (HC) animals were placed from the recording cages into regular home cages separately, and each REM sleep-deprived (RD) animal was placed on a round platform (diameter: 6.5 cm, surface was 0.5 cm above the water level) situated in the middle of a round water tank (diameter: 41 cm). The RD procedure was started at lights on and finished 72 h later when HC and RD rats were placed into the recording cages, and connected to the cables. All animals were kept undisturbed; food and water were available *ad libitum* during the whole period.

### Groups

Animals were randomly divided into four groups:

- Home Cage-Vehicle (HC-VEH; n = 6): 2 h polysomnographic recordings were made after spending 72 h in their own home cages; rats were treated chronically with vehicle.
- Home Cage-Escitalopram (HC-SSRI; n = 7): 2 h polysomnographic recordings made after spending 72 h in their own home cages; rats were treated chronically with escitalopram.
- REM sleep deprivation-Vehicle (RD-VEH; n = 7): 2 h polysomnographic recordings were made after spending 72 h on a small platform; rats were treated chronically with vehicle.
- REM sleep deprivation-Escitalopram (RD-SSRI; n = 7): 2 h polysomnographic recordings were made after spending 72 h on a small platform; rats were treated chronically with escitalopram.

### Sleep recording and scoring

Sleep recordings were started immediately after the RD procedure, during a 2 hour-long period starting at the onset of passive phase. EEG, EMG and motor activity were recorded as described earlier [41,46,47]. Rats were undisturbed throughout the recordings and had free access to standard rodent chow and tap water. Data were stored on computer for further analysis. The signals were amplified (Coulburn Lablinc System, USA; amplification factors approximately 5.000 for EEG and motor activity, 20.000 for EMG), conditioned by analogue filters (Coulburn Lablinc System, USA; filtering, below 0.50 Hz and above 60 Hz at 6 dB/octave), and subjected to analogue to digital conversion (MVRD-2200 V, Canopus, Japan) with a sampling rate of 128 Hz/channel. The digitized signals were displayed on a monitor and stored on the computer for further analysis. The vigilance states were scored visually for 4 s epochs as follows: active wakefulness (AW), the EEG is characterized by low amplitude activity at alpha (10–13 Hz) and beta (14–30 Hz) frequencies accompanied by high EMG and motor activity; passive wakefulness (PW), the EEG is characterized by low amplitude activity at alpha (10–13 Hz) and beta (14–30 Hz) frequencies accompanied by low EMG activity and motor activity; light slow-wave sleep (SWS-1), the EEG is characterized by high voltage slow cortical waves (0.5–4 Hz) interrupted by low voltage fast EEG activity (spindles 6–15 Hz) accompanied by reduced EMG and motor activity; deep slow-wave sleep (SWS-2), the EEG is characterized by high voltage (min. 350–400 μV) slow cortical waves (0.5-4 Hz) accompanied by reduced EMG and motor activity; intermediate stage (IS) of sleep, a brief stage just prior to REM sleep and sometimes just after it, characterized by unusual association of high-amplitude spindles (mean 12.5 Hz) and low-frequency (mean 5.4 Hz) theta rhythm; REM sleep, low amplitude and high frequency EEG activity with regular theta waves (5–9 Hz) accompanied by silent EMG and motor activity with occasional twitching. The polygraphic recordings were classified by sleep analysis software (SleepSign for Animal; Kissei Comtec America Inc., U.S.A.). Recordings were visually scored.

**Figure 1:**
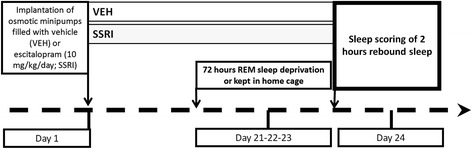
**Schematic illustration of the experimental design.**

The treatment schedules and the experimental protocol are graphically summarized in Figure 1.

### Modeling hypnograms as Markov chains

We modelled and compared hypnograms as time-continuous Markov chains. Such a modelling approach to characterize hypnograms has already been used by others [36–39,48], and more detailed mathematical background can be found there. In short, time-continuous Markov model assumes that the probability of the subsequent sleep stage depends only on the present stage and that the probability of moving from one stage to another exponentially increases with time. The rate of the changes is assumed to be constant and called transition rate. The primary interests are not transition rates themselves but rather how different they are compared to the control group. In the statistical literature the relative rates are called hazard ratios, but we feel that in the context of this paper this terminology would be confusing. Therefore we referred to hazard ratios as normalized transition rates (NTR); the reported rates are the relative rates compared to the HC-VEH group. If a NTR of a group is higher than 1, it indicates that the treatment increases the transition rate from one state to another compared to the control group. If a NTR is less than 1 the given treatment inhibits the transition. Instead of formal statistical tests we used the confidence intervals provided by the software to declare significance. If the confidence interval did not cover 1, we declared that the treatment effect is statistically significant at p < 0.05 level compared to control. Also, if the confidence intervals of two groups are not overlapping the two groups significantly differ at p < 0.05 level. There is another set of parameter of interest called sojourn time. Sojourn time is essentially the expected average time span of an event. Sojourn times have directly interpretable physical meanings and because of that, their absolute values are provided with the corresponding 95% confidence intervals. We declared that two sojourn times are statistically different if the corresponding 95% confidence intervals were not overlapping.

**Figure 2:**
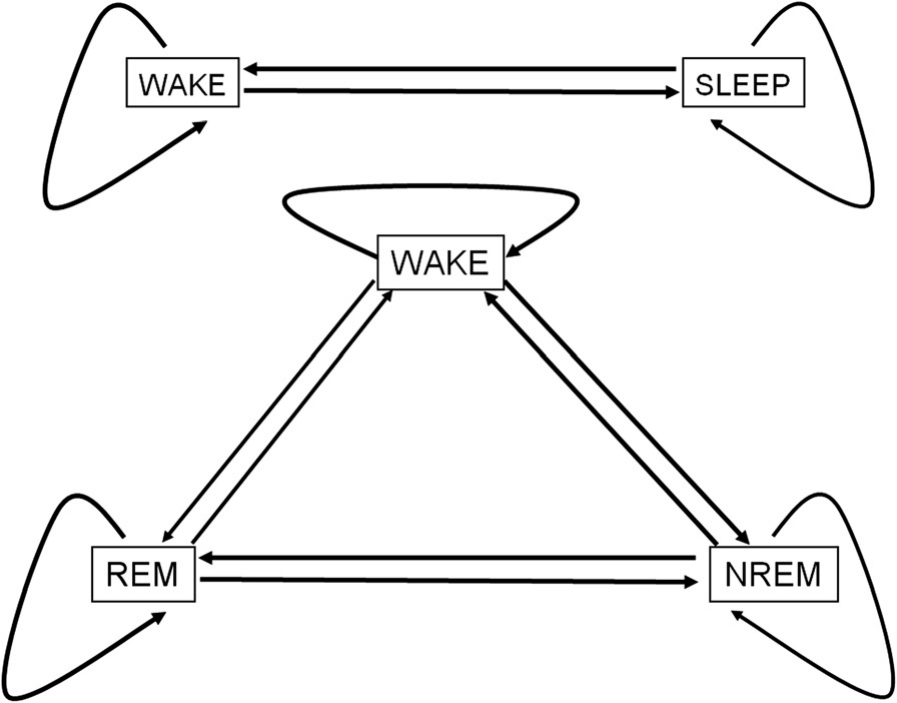
**Markov chain representations of sleep stages and transitions between them.** The system might jump to a new state or might remain in the current one at any time point. Straight lines represent the transition rates from and back between two stages and curved lines show staying in the current stage. The average time spent between two jumps in a given stage is the sojourn time of the stage. Four parameters (two transition rates and two sojourn times) are needed to describe a Markov model with two states (upper panel) while fitting a Markov model with three states (lower panel) requires the determination of nine parameters (six transition rates and three sojourn times).

Markov-modelling is a nonlinear regression modelling technique and it is based on numerical optimization. To achieve convergence, we faced two limitations of the applied software. First, it had a constraint that the transition rates are constant and independent from time. This limitation could have been overcome by visually segmenting the data into stationary periods, that is, when the observed stage frequencies were relatively constant. The time-segments were represented by an additional factor variable in the final statistical model. The effect of time has been factored out from the final statistical model so the reported sojourn times and NTRs are time independent estimates. The second limitation was that we could achieve successful convergence only with two or three stage models depicted in Figure 2. Keeping in mind these limitations we proceeded in the following way:

By visual inspection of the data, we split the whole observation period into an initial onset phase [starting at lights on, lasted about 30 minutes (0–2000 s)] and a subsequent steady phase [lasted about 90 minutes (2001–7200 s)] because of their markedly different sleep characteristics. In the onset phase, the frequency of wakeful periods showed a decreasing trend. In addition, since there were relatively few REM sleep periods in the onset phase, any attempt to fit a three-stage [WAKE, non-REM (NREM) and REM sleep] Markov model failed; the optimizing algorithm did not converge. To improve the numerical stability we merged the REM and NREM sleep stages into one (SLEEP), so the system had only two stages: WAKE and SLEEP. In contrast, the prevalence of each vigilance stages was constant in the steady phase, and the hypnogram showed much less non-stationary features than in the onset phase. So we could fit successfully a three-stage Markov model (WAKE, NREM and REM) without any further segmentation.

### Computation

We used the freely available R program [49] with the help of an R package called msn [50] for the Markovian analysis. All computations were run on a PC with 8 GB RAM and Intel Core-I5 2500 K processor.

### Standard sleep analysis

Vigilance parameters calculated in the present study were the following:

- Total time spent in WAKE (AW and PW stages), NREM (SWS-1, SWS-2 and IS stages), REM and SLEEP (NREM plus REM sleep) in the summarized first 2 h (1–2 h);
- Number of WAKE, NREM, REM and SLEEP episodes in 1–2 h [an episode was defined as a period of WAKE, NREM, REM and SLEEP lasting for ≥4 s (1 epoch)];
- Average duration of WAKE, NREM, REM and SLEEP episodes in 1–2 h;
- Sleep fragmentation: the sum of the number of awakenings (either AW or PW) that disconnected any sleep periods [51] in 1–2 h;
- REM sleep latency: the time elapsed from the start of sleep until the first occurrence of REM sleep;
- Sleep (SWS-1) latency: the time elapsed between light onset and the first occurrence of SWS-1;
- First REM item: the length of the first uninterrupted REM and IS sleep period (after the first REM epoch any IS epochs are permitted till the end of the REM sleep item);

### EEG power spectral analysis [quantitative EEG (Q-EEG)]

EEG power spectra were computed for consecutive 4 s epochs in the frequency range 1.25 to 60 Hz (fast Fourier transformation routine, Hanning window; frequency resolution, 0.25 Hz; the range 0.5-1 Hz were omitted from the analysis). Epochs with artefacts were discarded on the basis of the polygraph records. Adjacent 0.25-Hz bins were summed into 1-Hz bins, and those above 60 Hz were omitted. Bins are marked by their upper limits, thus, 2 Hz refers to 1.25 to 2.00 Hz [46]. Frequencies were binned into delta (1–4 Hz), theta (5–9 Hz), alpha (10–13 Hz), beta (14–30 Hz) and gamma (31–60 Hz) power bands. The power values of consecutive 4 s EEG epochs in AW, PW, SWS-1, SWS-2, IS and REM sleep were separately averaged in the summarized first 2 h of sleep.

### Statistical hypothesis testing

Vigilance data were subjected to two-way analysis of variance (ANOVA) with two main factors: treatment (VEH or SSRI) and rebound (HC or RD) followed by Tukey’s honest significant difference test.

The EEG power spectra were evaluated by three-way repeated measures ANOVA with three main factors [treatment (VEH or SSRI), rebound (HC or RD) and frequency bins repeated] on theta (5–9 Hz) and alpha (10–13 Hz) frequency bands in SWS-1, respectively, followed by Tukey’s honest significant difference test. In SWS-2, three-way repeated measures ANOVA was used with three main factors [treatment (VEH or SSRI), rebound (HC or RD) and theta frequency bins (5–9 Hz), repeated] followed by Tukey’s honest significant difference test.

The sleep and Q-EEG analyses were evaluated for the summarized first 2 h. *P* values less than 0.05 (*p* < 0.05) were defined as statistically significant. Data in all figures are presented as mean ± SEM of 6–7 animals per group. For statistical analysis and graphs, Prism 6 (GraphPad Software, Inc., USA) software was used.

## Results

### Markov chain analysis

#### Onset phase (0–2000 s)

**Figure 3:**
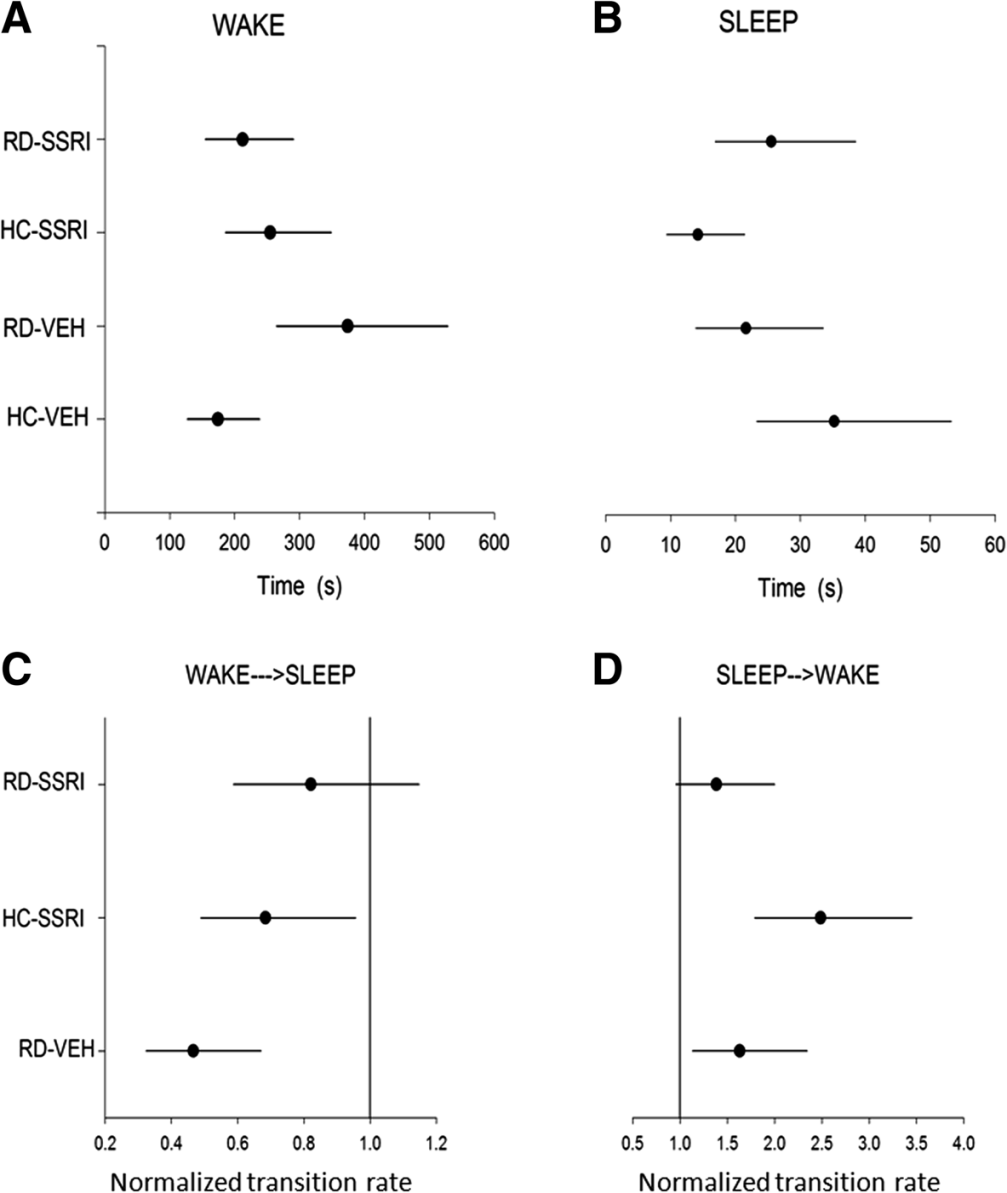
**Sojourn times and normalized transition rates in the onset phase (0–2000 s; ca. first 30 min).** The estimated mean of the average time spent between two jumps (sojourn time; s) in **A)** WAKE and **B)** SLEEP, and the normalized transition rates (relative rates compared to the transition rate of HC-VEH group, which is 1) **C)** from WAKE to SLEEP and **D)** from SLEEP to WAKE states. Each point (●) represents the mean values and the line segments show the 95% confidence interval surrounding them. Groups: home cage-chronic vehicle treatment (HC-VEH); home cage-chronic escitalopram treatment (HC-SSRI); REM sleep deprivation-chronic vehicle treatment (RD-VEH); REM sleep deprivation-chronic escitalopram treatment (RD-SSRI).

**Figure 4:**
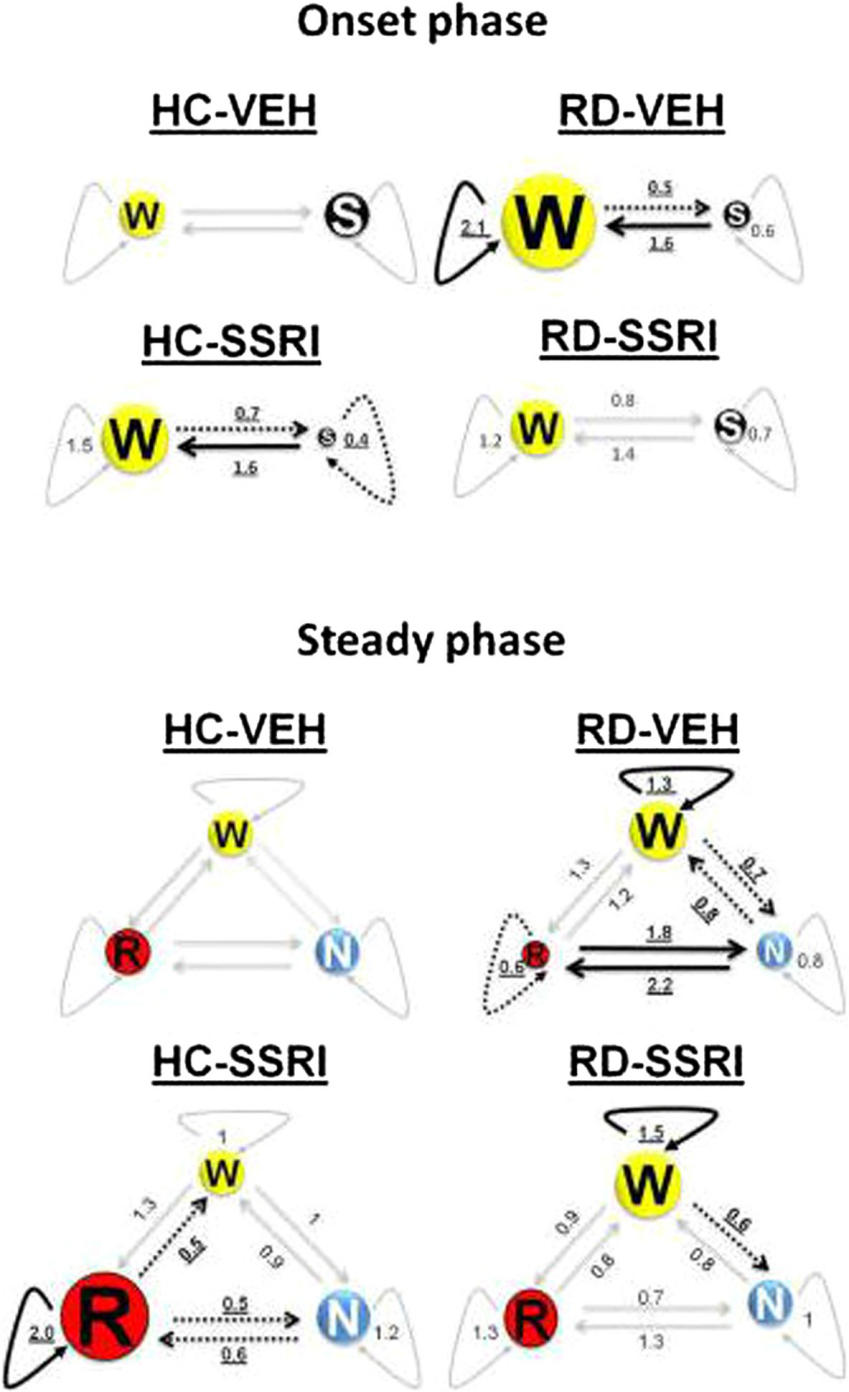
**Illustrations of Markov chains during the onset and steady phase.** Numeric data represent the relative mean values of sojourn times and transition rates in the treatment groups compared to the mean values of the HC-VEH group (sojourn times and transition rates of the HC-VEH group are 1). The size of circles aligns the changes in sojourn times. Thick, uninterrupted arrows represent significant increase, dashed arrows show significant decrease and slight arrows sign no significant changes in transition speed compared to the HC-VEH group. Significant alterations are highlighted in bold, underlined characters.

Sojourn times and transition rates during the onset phase are depicted in Figure 3; numerical values and statistically significant changes are represented in Table 1. Schematic illustration of sojourn times and transition rates in the treatment groups relative to the HC-VEH group is shown in Figure 4.

**Table 1:** **Sojourn times and Normalized Transition Rates (NTR) in the onset phase**

**Onset phase (starting at light onset; 0–2000 s)**						
**Group**	**WAKE**			**SLEEP**		
	**Sojourn time (s)**	**L**	**U**	**Sojourn time (s)**	**L**	**U**
**RD-SSRI**	212.102	155.104	290.046	25.467	16.864	38.460
**HC-SSRI**	254.713	186.264	348.315	**14.176** ^*^	9.387	21.408
**RD-VEH**	**374.026** ^*^	264.968	527.973	21.609	13.941	33.493
**HC-VEH**	174.131	127.337	238.122	35.239	23.334	53.216
	**WAKE → SLEEP**			**SLEEP → WAKE**		
	**NTR**	**L**	**U**	**NTR**	**L**	**U**
**RD-SSRI**	0.821	0.587	1.147	1.384	0.958	1.999
**HC-SSRI**	**0.684** ^*^	0.489	0.956	**2.486** ^*^	1.791	3.450
**RD-VEH**	**0.465** ^*^	0.323	0.670	**1.631** ^*^	1.135	2.343

Sojourn time: the estimated mean of the average time span of an event;

Normalized Transition Rate (NTR): relative transition rate compared to the HC-VEH group (NTR of the HC-VEH group is 1);

* *p* < 0.05, significant effect compared to the HC-VEH group;

L and U: lower and the upper limits of the corresponding 95% confidence interval, respectively;

Normalized Transition Rate (NTR): relative transition rate compared to the HC-VEH group (NTR of the HC-VEH group is 1);

Groups: home cage plus chronic vehicle treatment (HC-VEH); home cage plus chronic escitalopram treatment (HC-SSRI); REM sleep deprivation plus chronic vehicle treatment (RD-VEH); REM sleep deprivation plus chronic escitalopram treatment (RD-SSRI).

In overall, separately, both chronic escitalopram treatment and RD increased wakefulness; however, when the two interventions were applied together, these effects did not add but rather antagonized each other.

In detail, chronic escitalopram (HC-SSRI group) decreased the SLEEP sojourn time (by about 60%; Figure 3B), while RD (RD-VEH group) increased the WAKE sojourn (by more than 100%; Figure 3A) compared to control, because their confidence intervals do not overlap with that of the HC-VEH group. In addition, the process falling into sleep was decelerated in both the HC-SSRI and RD-VEH groups (decreased NTRs of WAKE → SLEEP; Figure 3C) and waking up was promoted (increased NTRs of SLEEP → WAKE; Figure 3D). The confidence intervals do not cover 1 (the NTR of HC-VEH group is 1), so the effect is significant at p < 0.05 level. The RD-SSRI group did not cause any significant difference in transition rates compared to any other groups.

#### Steady phase (2001–7200 s)

**Figure 5:**
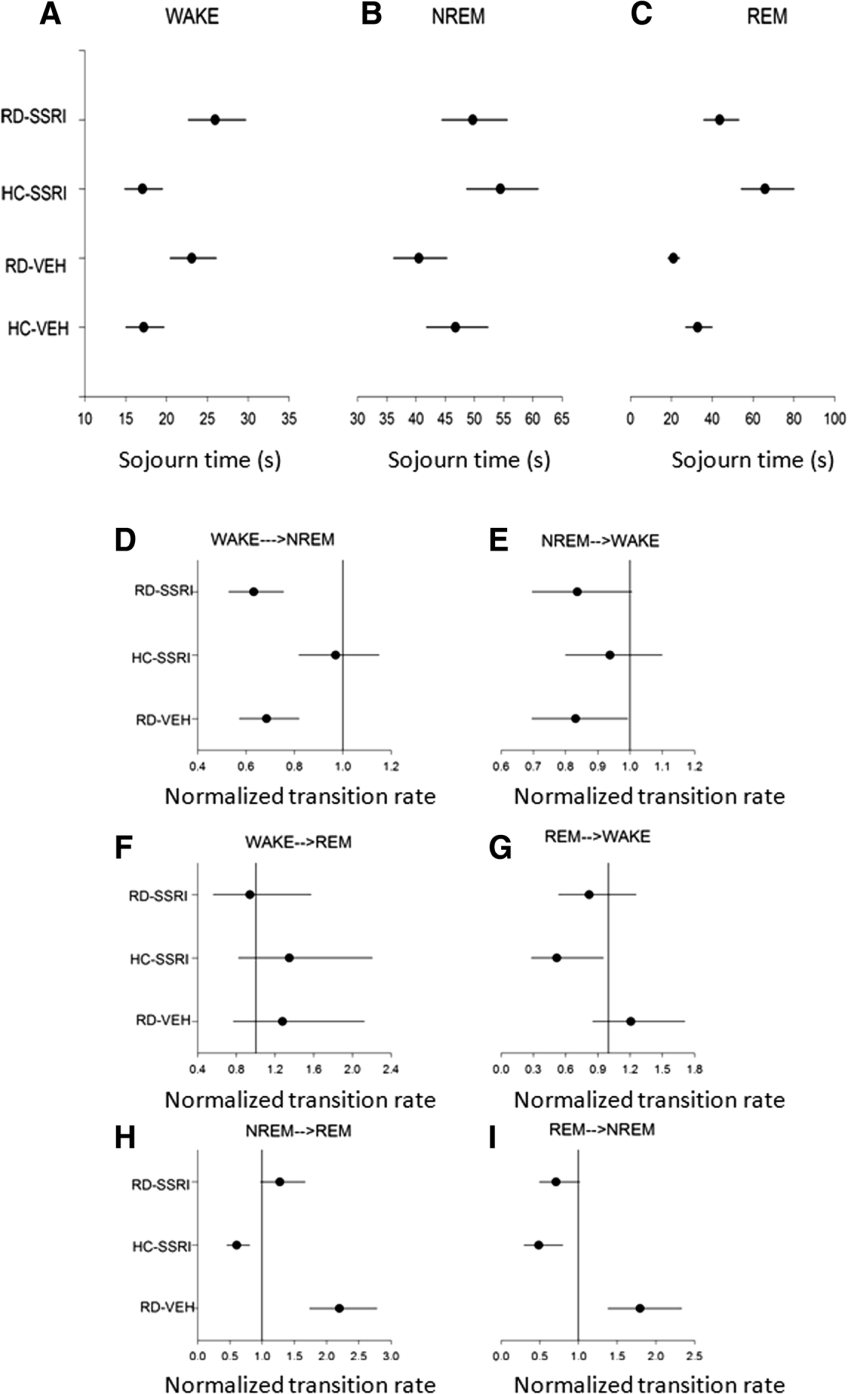
**Sojourn times and normalized transition rates in the steady phase (2001–7200 sec; ca. 30–120 min).** The estimated mean of the average time spent between two jumps (sojourn time; s) in **A)** WAKE, **B)** NREM and **C)** REM sleep, and the normalized transition rates (relative rates compared to the transition rate of HC-VEH group, which is 1) **D)** from WAKE to NREM, **E)** from NREM to WAKE, **F)** from WAKE to REM, **G)** from REM to WAKE, **H)** from NREM to REM **I)** and from REM to NREM states. Each point (●) represents the mean values and the line segments show the 95% confidence interval surrounding them. Groups: home cage-chronic vehicle treatment (HC-VEH); home cage-chronic escitalopram treatment (HC-SSRI); REM sleep deprivation-chronic vehicle treatment (RD-VEH); REM sleep deprivation-chronic escitalopram treatment (RD-SSRI).

Sojourn times and transition rates during the steady phase are depicted in Figure 5; numerical values and statistically significant changes are represented in Table 2. Schematic illustration of sojourn times and transition rates in the treatment groups relative to the HC-VEH group is shown in Figure 4.

**Table 2:** **Sojourn times and Normalized Transition Rates (NTR) in the steady phase**

**Steady phase (2001–7200 s)**									
**Group**	**WAKE**			**NREM**			**REM**		
	**Sojourn time (s)**	**L**	**U**	**Sojourn time (s)**	**L**	**U**	**Sojourn time (s)**	**L**	**U**
**RD-SSRI**	**25.939** ^*#^	22.668	29.681	49.719	44.440	55.625	**43.633** ^**#&**^	35.921	53.001
**HC-SSRI**	17.051	14.901	19.511	54.452	48.670	60.921	**65.816** ^*^	54.183	79.946
**RD-VEH**	**23.081** ^*^	20.442	26.061	40.459	36.157	45.273	**20.999** ^*^	18.379	23.994
**HC-VEH**	17.199	15.031	19.681	46.758	41.793	52.312	32.844	27.039	39.896
	**WAKE → NREM**			**NREM → WAKE**					
	**NTR**	**L**	**U**	**NTR**	**L**	**U**			
**RD-SSRI**	**0.631** ^*#^	0.528	0.755	0.836	0.696	1.005			
**HC-SSRI**	0.970	0.819	1.150	0.938	0.800	1.099			
**RD-VEH**	**0.685** ^*^	0.573	0.819	**0.831** ^*^	0.695	0.993			
	**WAKE → REM**			**REM → WAKE**					
	**NTR**	**L**	**U**	**NTR**	**L**	**U**			
**RD-SSRI**	0.940	0.562	1.573	0.820	0.535	1.255			
**HC-SSRI**	1.347	0.822	2.206	**0.518** ^*^	0.282	0.951			
**RD-VEH**	1.277	0.768	2.123	1.208	0.853	1.710			
	**NREM → REM**			**REM → NREM**					
	**NTR**	**L**	**U**	**NTR**	**L**	**U**			
**RD-SSRI**	**1.274** ^**#&**^	0.975	1.665	**0.709** ^**&**^	0.497	1.013			
**HC-SSRI**	**0.605** ^*^	0.453	0.806	**0.486** ^*^	0.297	0.796			
**RD-VEH**	**2.198** ^*^	1.739	2.779	**1.796** ^*^	1.382	2.334			

Sojourn time: the estimated mean of the average time span of an event;

Normalized Transition Rate (NTR): relative transition rate compared to the HC-VEH group (NTR of the HC-VEH group is 1);

L and U: lower and the upper limits of the corresponding 95% confidence interval, respectively;

* *p* < 0.05, significant effect compared to the HC-VEH group;

^**#**^ *p* < 0.05, significant effect compared to the HC-SSRI group;

^&^ *p* < 0.05, significant effect compared to RD-VEH group;

Groups: home cage plus chronic vehicle treatment (HC-VEH); home cage plus chronic escitalopram treatment (HC-SSRI); REM sleep deprivation plus chronic vehicle treatment (RD-VEH); REM sleep deprivation plus chronic escitalopram treatment (RD-SSRI).

In overall, chronic escitalopram treatment attenuated the RD-caused decrease in REM sojourn and the accelerated transitions between NREM and REM stages. However, the SSRI had no influence on the elevated WAKE pressure caused by RD.

In detail, the REM sleep residence time (Figure 5C) was significantly decreased in the RD-VEH group, and the transition rates from NREM to REM sleep and in the backward direction (NREM↔REM, Figure 5H-I) were increased compared to the cage control (HC-VEH group). On the contrary, the chronic escitalopram treatment alone (HC-SSRI group) doubled the length of REM sleep sojourn (Figure 5C) and slowed down the NREM↔REM transitions (Figure 5H-I) as well as the REM → WAKE transitions (Figure 5G) compared to the HC-VEH group. Regarding the RD-SSRI group, increased REM sojourn (Figure 5C) and decreased NREM↔REM transitions (Figure 5H-I) were found compared to the RD-VEH group. In addition, the rate of NREM → REM transitions (Figure 5H) was significantly accelerated compared to the SSRI-treated cage control (HC-SSRI group).

Furthermore, WAKE sojourn time (Figure 5A) was significantly enhanced after RD either with or without SSRI treatment (RD-VEH vs. HC-VEH; RD-SSRI vs. HC-SSRI and HC-VEH groups). In line with the increased WAKE span, falling into sleep (WAKE → NREM, Figure 5D) was decelerated after RD (RD-VEH vs. HC-VEH groups and RD-SSRI vs. HC-SSRI and HC-VEH groups). Additionally, the RD-VEH group significantly decreased also the NREM → WAKE transition speed (Figure 5E) compared to the cage control (HC-VEH group).

### Standard sleep analysis

#### Vigilance parameters

##### REM sleep parameters

**Figure 6:**
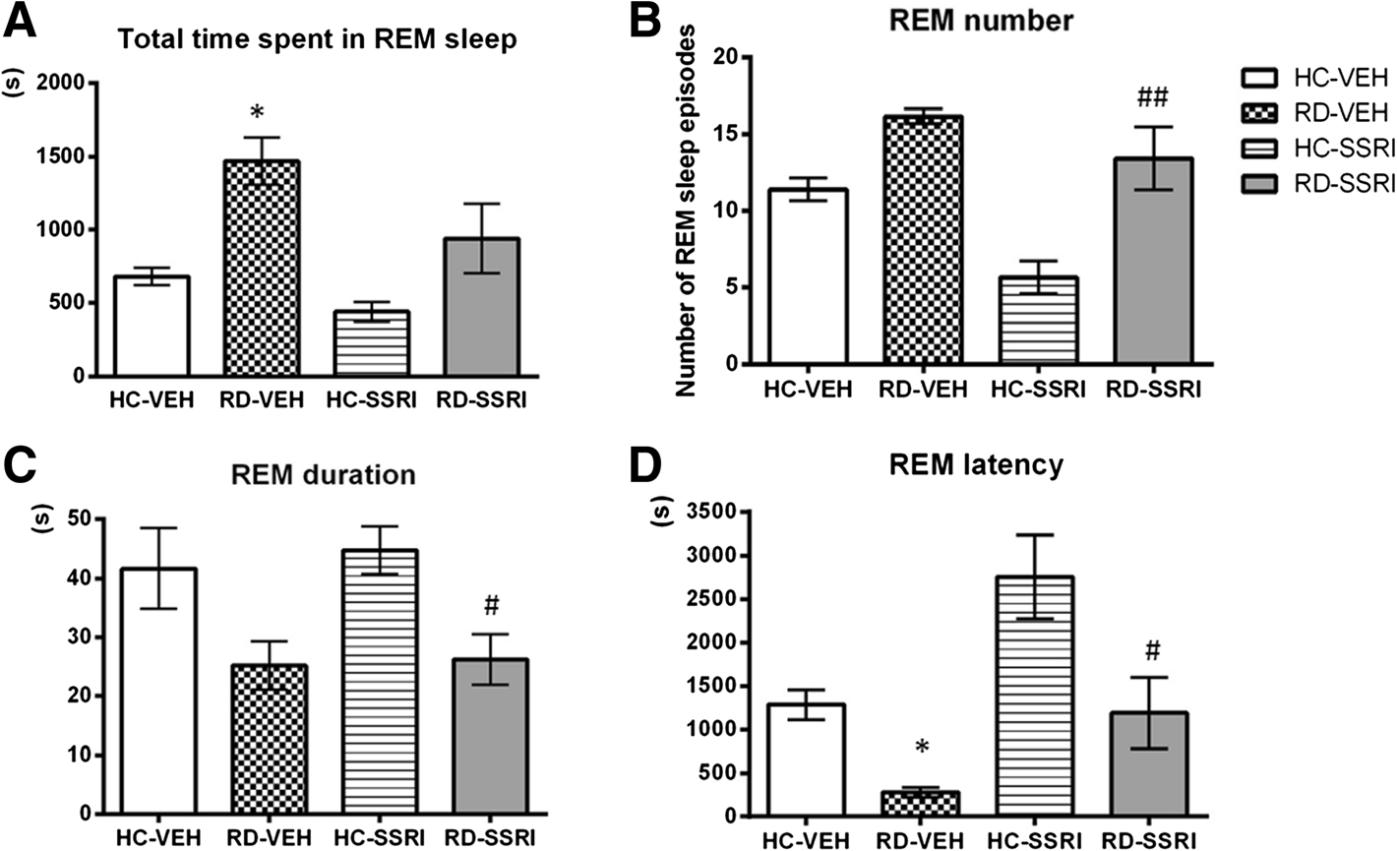
**Aggregated REM sleep measures calculated by standard sleep analysis in the summarized first 2 h of passive phase.** Changes in the **A)** amount, **B)** episode number, **C)** episode duration and **D)** the latency time of REM sleep. Data are presented as mean ± SEM of 6–7 animals per group. *p < 0.05 means significant post hoc effects of RD-VEH group compared to the HC-VEH group; ^#^ *p* < 0.05 and ^##^ *p* < 0.01 mean significant post hoc effects of and RD-SSRI group compared to HC-SSRI group. Groups: home cage-chronic vehicle treatment (HC-VEH); home cage-chronic escitalopram treatment (HC-SSRI); REM sleep deprivation-chronic vehicle treatment (RD-VEH); REM sleep deprivation-chronic escitalopram treatment (RD-SSRI).

Based on two-way ANOVA statistics (Table 3), RD and chronic SSRI treatment had marked effects in the total time spent in REM, REM number and REM latency parameters (Figure 6A,B and D, respectively). The 3-day-long RD by itself (RD-VEH group) caused a marked REM rebound by means of increased time spent in REM sleep (Figure 6A) and decreased REM onset latency time (Figure 6D) compared to the HC-VEH group (for significant Tukey’s post hoc comparisons see Table 3). Additionally, the RD-SSRI group significantly decreased the REM episode duration (Figure 6C) compared to the cage control (for significant two-way ANOVA and Tukey’s post hoc comparisons see Table 3).

**Table 3:** **ANOVA results and mean ±SEM values for vigilance parameters in the summarized first 2 h**

		**Two-way ANOVA**						**Mean ± SEM**			
		**Treatment effect**		**Rebound effect**		**Treatment x Rebound**					
		**F**_**1,23**_	**p**	**F**_**1,23**_	**p**	**F**_**1,24**_	**p**	**HC-VEH**	**RD-VEH**	**HC-SSRI**	**RD-SSRI**
**Total time spent in stage**	**WAKE**	0.97	0.3337	2.00	0.1703	0.89	0.3529	2063.0 ± 208.2	2991.0 ± 337.2	2823.0 ± 444.4	3006.0 ± 473.7
	**NREM sleep**	0.0008	0.9772	11.08	**0.0029**	1.14	0.2963	4325.0 ± 152.8	**2921.0 ± 195.7** ^*^	3975.0 ± 430.1	3253.0 ± 367.8
	**REM sleep**	9.18	**0.0066**	15.85	**0.0007**	0.10	0.7502	680.8 ± 58.8	**1470.0 ± 160.9** ^*^	401.7 ± 69.0	940.6 ± 236.3
	**SLEEP**	0.97	0.3335	2.00	0.1706	0.89	0.3527	5136.6 ± 208.2	4209.1 ± 337.2	4376.5 ± 444.8	4193.7 ± 473.7
**Number of stage episodes**	**WAKE**	1.45	0.2393	2.03	0.1673	0.08	0.7757	44.5 ± 5.3	39.2 ± 4.5	40.2 ± 3.8	32.4 ± 4.5
	**NREM sleep**	0.96	0.3354	0.08	0.7717	<0.0001	0.9924	71.5 ± 16.5	74.8 ± 9.5	60.7 ± 6.1	63.8 ± 11.2
	**REM sleep**	7.56	**0.0117**	25.22	**<0.0001**	0.71	0.4071	11.4 ± 0.7	16.1 ± 0.4	5.0 ± 1.1	**13.43 ± 2.0** ^**##**^
	**SLEEP**	0.63	0.4330	1.71	0.2035	0.26	0.6119	71.5 ± 17.3	53.1 ± 7.5	58.2 ± 6.2	50.2 ± 7.9
**Average duration of stage episodes**	**WAKE**	0.75	0.3925	2.84	0.1024	0.15	0.7013	34.6 ± 6.0	64.5 ± 14.8	52.6 ± 12.2	71.4 ± 18.8
	**NREM sleep**	0.08	0.7756	5.74	**0.0250**	0.65	0.4284	74.27 ± 14.0	43.5 ± 7.1	69.3 ± 11.0	54.0 ± 4.7
	**REM sleep**	0.19	0.6664	13.49	**0.0015**	0.04	0.8299	41.6 ± 6.8	25.1 ± 4.1	44.8 ± 4.0	**26.2 ± 4.2** ^**#**^
	**SLEEP**	0.05	0.8139	0.24	0.6229	0.09	0.7580	88.8 ± 16.4	91.8 ± 18.3	79.9 ± 12.2	93.0 ± 16.91
**Sleep fragmentation**		0.64	0.4308	1.72	0.2022	0.28	0.5997	71.0 ± 17.4	52.4 ± 7.4	57.5 ± 6.2	49.7 ± 7.8
**REM sleep latency**		5.05	**0.0359**	16.53	**0.0006**	0.10	0.7519	1285.6 ± 171.6	**280.0 ± 57.1** ^*^	2755.4 ± 480.7	**1187.4 ± 410.7** ^**#**^
**Sleep (SWS-1) latency**		0.06	0.7992	0.38	0.5385	2.54	0.1244	820.7 ± 189.5	1321.0 ± 229.3	1122 ± 257.1	903.4 ± 206.9
**First REM item**		0.30	0.5848	0.15	0.7005	0.50	0.4860	78.4 ± 17.2	102.0 ± 24.5	81.7 ± 20.5	74.8 ± 20.4

Significant effects of two-way ANOVA statistics are highlighted in bold.

* *p* < 0.05 indicates significant Tukey’s post hoc comparisons compared to the HC-VEH group; ^#^ *p* < 0.05 and ^##^ *p* < 0.01 represent significant Tukey’s post hoc comparisons compared to the HC-SSRI group; Data represent mean **±** SEM values of 6–7 animals.

Mean values indicate time (seconds) in the cases of total time spent in stages, average duration of stages, REM sleep latency (the time elapsed from the start of sleep until the first occurrence of REM sleep), sleep (SWS-1) latency (the time elapsed between light onset and the first occurrence of SWS-1) and first REM item (the length of the first uninterrupted REM and IS sleep period). Means are numbers in the cases of number of stage episodes (at least 4 sec long) and sleep fragmentation (the sum of the number of AW and PW episodes that disconnected any sleep periods).

Groups: home cage plus chronic vehicle treatment (HC-VEH); home cage plus chronic escitalopram treatment (HC-SSRI); REM sleep deprivation plus chronic vehicle treatment (RD-VEH); REM sleep deprivation plus chronic escitalopram treatment (RD-SSRI).

Chronic escitalopram treatment alone (HC-SSRI group) did not cause any significant changes. Moreover, it seems that the SSRI did not attenuate markedly the REM sleep rebound, since there was no significant interaction between the effects of RD and chronic escitalopram treatment in any REM sleep parameters, and RD could provoke REM rebound even besides SSRI administration [the RD-SSRI group enhanced the REM number (Figure 6B) and decreased the REM latency (Figure 6D) compared to the HC-SSRI group, Table 3].

##### NREM sleep parameters

The RD-VEH group decreased the total time spent in NREM sleep compared to the cage control (for significant rebound effect and Tukey’s post hoc comparison see Table 3). In addition, the rebound caused a decreasing effect in NREM sleep episode duration (Table 3) without any significant post hoc difference between the groups.

Similarly to REM sleep, escitalopram did not influence the effect of RD on NREM sleep (no significant rebound by treatment interaction, Table 3).

There was no significant alteration in the following vigilance parameters: total time spent in WAKE and SLEEP, number of WAKE, NREM sleep and SLEEP episodes, average duration of WAKE and SLEEP episodes, sleep fragmentation, sleep (SWS-1) and SWS-2 latencies as well as in the first REM item. ANOVA results and mean ± SEM values assigned with all significant Tukey’s post hoc comparisons are summarized in Table 3.

#### Quantitative EEG (Q-EEG) spectra

**Figure 7:**
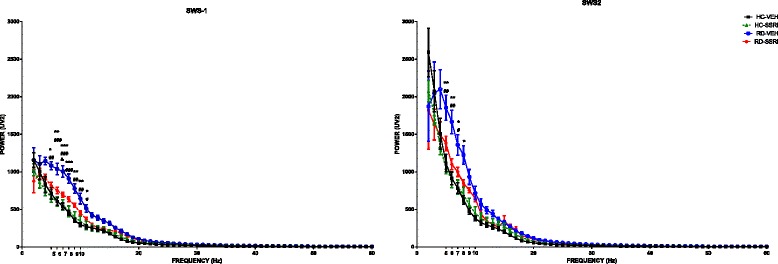
**EEG spectra during slow-wave sleep stages.** Effects of chronic SSRI treatment, 72 h of REM sleep deprivation and their combination on the power density of theta (5–9 Hz) and alpha (10–13 Hz) frequencies during light slow-wave sleep (SWS-1) and on theta frequencies during deep slow-wave sleep (SWS-2). Each point represents mean ± SEM values of 1-Hz bins in a 2 h recording period of 6–7 animals. *p < 0.05, **p < 0.01 and ***p < 0.05 mean significant post hoc effects of RD-VEH group compared to the HC-VEH group; ^#^ *p* < 0.05, ^##^ *p* < 0.01 and ^###^ *p* < 0.001 mean significant post hoc effects of RD-VEH group compared to HC-SSRI group, and & *p* < 0.05 represents significant post hoc effect of RD-SSRI group compared to RD-VEH group. Groups: home cage plus chronic vehicle treatment (HC-VEH); home cage plus chronic escitalopram treatment (HC-SSRI); REM sleep deprivation plus chronic vehicle treatment (RD-VEH); REM sleep deprivation plus chronic escitalopram treatment (RD-SSRI).

RD by itself (RD-VEH group) caused a marked elevation on the theta (5–9 Hz) power density during both SWS-1 and SWS-2 (rebound effect: *F*_1,23_ = 29.92, *p* < 0.0001 *and F*_3,23_ = 21.33, *p* < 0.001, respectively) compared to the HC-VEH and the HC-SSRI groups (significant Tukey’s post hoc comparisons in SWS-1 and SWS-2 are presented in Figure 7). Chronic SSRI administration avoided this effect of RD since theta wave activity in the RD-SSRI group was attenuated (SWS-1: treatment effect: *F*_1,23_ = 6.42, *p* < 0.05; treatment x rebound interaction: *F*_1,23_ = 6.94, *p* < 0.05; significant Tukey’s post hoc comparison at 7 Hz between the RD-VEH and RD-SSRI groups, Figure 7; SWS-2: treatment effect: *F*_1,23_ = 4.25, *p* = 0.0506; treatment x rebound interaction: *F*_1,23_ = 4.93, *p* = 0.0364; frequency x treatment interaction: *F*_4,92_ = 4.93, p < 0.01; frequency x rebound interaction *F*_4,92_ = 3.66, p < 0.01).

In addition, REM sleep deprivation (RD-VEH group) caused a significant elevation in the power density of alpha frequency band (10–13 Hz) in SWS-1 [rebound effect: *F*_1,23_ = 13.64, *p* < 0.05, significant Tukey’s post hoc comparisons between RD-VEH vs. HC-VEH and RD-VEH vs. HC-SSRI groups at 10 and 11 Hz, Figure 7], which was interfered by chronic SSRI treatment (treatment x rebound interaction: *F*_1,23_ = 4.35, *p* < 0.05; frequency x rebound interaction: *F*_3,69_ = 18.45, *p* = 0.0000).

There were no significant changes in the EEG spectra during AW, PW, IS and REM sleep at any frequencies studied.

## Discussion

In this study we demonstrated accelerated transitions between NREM and REM sleep stages and shortened REM sleep sojourn after 3-day-long RD applying the flower pot method. This REM sleep fragmentation returned to the normal level by SSRI co-treatment, however, analysis of the aggregate sleep measures revealed that chronic escitalopram did not influence the REM rebound markedly. In addition, escitalopram avoided the elevated wake pressure during the first 30 min of the rebound period, but not later, when falling asleep remained inhibited. The Q-EEG spectra showed that escitalopram attenuated the elevated theta power density during the SWS stages after RD.

### Effects of chronic escitalopram treatment on REM and NREM sleep after REM sleep deprivation

Markov-chain analysis showed that RD alone led to REM sleep fragmentation (decreased REM sojourn; doubled transitions between NREM and REM stages). On the contrary, chronic SSRI treatment elongated the REM sleep episodes (increased REM sojourn; inhibited traffic between NREM and REM sleep) in home cage animals and reduced the accelerated REM transition processes after RD (Figure 5C, H-I, Table 2). The standard descriptive REM sleep parameters, REM episode number and duration (Figure 6B-C), tended to change in the same directions, however, these changes were not significant.

Concerning REM sleep, the pontine cholinergic (REM-on) neurons are essential component of its generation, and are inhibited reciprocally by monoaminergic (REM-off) neurons including the serotonergic cell groups [52]. After RD, higher cholinergic and lower serotonergic neuronal activities were found [53,54]. Since a large body of evidence indicates that major depression is associated with an increase in cholinergic and a decrease in serotonergic neurotransmission, it has been proposed that this imbalance would be responsible for the disinhibition of REM sleep in depressed patients [25]. In accordance with our results found after RD, chronic mild stress for 21 days induced an increase in the number of transitions into REM sleep [55]. In addition, previous data from our laboratory point out that although REM sleep rebound itself is caused by the sleep deficit, the fragmentation of REM sleep might be the consequence of the sub-chronic stress caused by the flower pot method [11]. We can assume that the accumulated homeostatic need for REM sleep after RD might gradually shift the relative balance of mutual inhibition between REM-on and REM-off cells, and could be responsible for the accelerated REM sleep transitions. In addition, the reverse transition processes found after the SSRI treatment may be the result of opposite changes in the central serotonergic tone which tend to increase after escitalopram and decrease during the rebound sleep. Since altered REM transitions returned to the normal level after escitalopram, we can speculate that the serotonergic-cholinergic imbalance after RD might be compensated by chronic dosing of SSRI.

Regarding aggregate sleep measures, the standard sleep analysis showed that RD caused an elevated REM sleep time by means of moderately increased REM number, and shortened REM sleep latency; additionally, it attenuated the NREM sleep time (Figure 6, Table 3). These results are consistent with previous reports [11,56]. Several studies have suggested earlier that increased REM number and reduced REM latency may be the consequence of nonspecific effects of stress [11,32,55,57]. Furthermore, stress could be a main factor also in the suppression of NREM sleep [58–60]. Some studies have revealed also an enhancement in REM episode duration (in the first 3 h [12] and 2–24 h of the recovery period after 72 h RD [11]; in the first 6 h after 2 days of 18 h RD [56]). However, we did not find here an enhanced REM duration after RD; in fact, there was a tendency to decrease regarding this parameter. Since a decrease in REM sleep duration has also been shown after intracerebroventricular injection of corticotropin-releasing hormone [60], it is possible that stress can negatively affect REM episode duration. In addition, the increment in this parameter could be the specific consequence of RD and not that of stress. Considering the abovementioned results, it is likely that during the first 2 h of rebound, stress may play the main role in sleep regulation. Furthermore, we cannot neglect the possibility that the time interval examined here might be too short to manifest marginal effects of RD on REM duration. Another possible cause of this discrepancy could be that in some studies short REM attempts (a period of REM sleep lasting for ≥16 s and not interrupted by ≥16 s of other vigilance state) were excluded from REM sleep episodes [11,12]. So, we evaluated our data also according to the definition above and we found the similar effect of RD on REM duration, but it was only a tendency to increase (data not shown). This result can confirm that short REM attempts markedly influence the results of average duration of REM sleep episodes after RD.

As regards NREM sleep, we have to mention that during small platform RD, beyond REM sleep, a considerable amount of NREM sleep might also be deprived which can be manifested in NREM sleep rebound during the recovery [61]. We demonstrated earlier that rats spent significantly more time in SWS after the 3-day-long flower pot procedure in the active phase of the recovery day. However during the passive phase, immediately after RD, a decrease in SWS sleep time was found [11], which is in agreement with our present results.

Escitalopram by itself caused only minor changes in REM sleep parameters which are in accordance with previous findings (see in review: [62]). Similarly to the effect of RD alone, our results indicated that combination of RD and chronic escitalopram also caused a REM rebound by means of increased REM number and decreased REM latency (Figure 6B,D). We reported earlier that acute escitalopram administration (10 mg/kg, i.p.) in rats immediately after the same RD protocol attenuated the REM sleep rebound (decreased time spent in REM and REM number, increased REM latency) during the first 3 h of recovery sleep [12]. These results provided evidence that acute escitalopram is still able to reduce REM sleep despite the strong REM pressure. Considering our present findings, this REM rebound-reducing effect of escitalopram after RD diminishes over chronic dosing. Previous studies indicated that the typical REM-reducing effect of SSRI antidepressants attenuates during the chronic administration, and REM sleep amount returns towards baseline (see in review: [62]). This process has been suggested to be mediated mainly by the adaptation of postsynaptic serotonin (5HT)1A receptors [63]. The desensitization of somatodendritic 5-HT1A autoreceptors could also be responsible for the therapeutic effects of both chronic SSRI treatment [64–66] and SD therapies [26,67,68], at least in part.

### Effects of chronic escitalopram treatment on wakefulness after REM sleep deprivation

The RD alone weakened the condition of stable sleep resulted in more frequent awakenings during the first 30 min (onset phase). This change manifested in elongated WAKE sojourn time, because the transitions to SLEEP were inhibited and the rate of awakenings from SLEEP was increased. The effects of chronic escitalopram treatment on the SLEEP↔WAKE transitions were qualitatively the similar to the changes after RD. Interestingly, when the two interventions were applied together (RD-SSRI group), these effects were not added as might be expected, but rather antagonized each other (Figure 3, Table 1).

In accordance with our results, during the initial phase of sleep rebound after long-term RD (72–96 h), animals stay awake for approximately 30 min, when aggressiveness, hyperactivity, irritability and hypersexuality were noticed, suggesting that this condition may be used to investigate mechanisms of stress-induced insomnia [69]. Several studies suggested that the hypothalamic-pituitary-adrenal (HPA) axis activation could be responsible for the wake enhancement under stress exposure [58,70]. Citalopram treatment for 5 days, starting 2 days before 72 h RD using the flower pot method reversed the anxiety-like behaviour in mice after the deprivation [19]. Furthermore, chronic administration of several antidepressants reduced the responses of HPA axis activity in depressed humans and rats exposed to chronic stress [71–74]. Therefore, our results indicate that chronic antidepressant medication may suppress the elevated wake pressure after RD possibly due to the reduced stress level.

In contrast to the onset phase, escitalopram treatment did not influence the elevated wake pressure during the further 90 min (called steady phase) of rebound sleep, which manifested in increased WAKE sojourn time and decelerated transitions from WAKE to NREM sleep (Figure 5A,D, Table 2). The possible cause of this discrepancy between the effects of escitalopram in the onset and steady phase is that wake promotion can be mainly the consequence of stress during the initial phase, but later the influence of RD might dominate. However, due to the limitations of this study (e.g. absence of large platform as stress control or measuring behaviour), firm conclusion cannot be drawn. Furthermore, beyond the serotonergic neurotransmission, RD influences other monoaminergic systems (see in review: [75]) involved in wake generation, and cannot be influenced by the changes caused by chronic SSRI administration.

Based on the aggregate sleep measures (Table 3), we did not find any changes denoting enhanced wake pressure or sleep fragmentation after RD, which is consistent with previous reports [11,12,76].

### Effects of chronic escitalopram treatment on theta wave activity during slow-wave sleep after REM sleep deprivation

The spectral analysis of sleep established robust increases in theta frequency power caused by RD during both SWS-1 and SWS-2 stages, which effects were prevented by chronic escitalopram administration (Figure 7).

In accordance with our results, enhanced theta activity in NREM sleep has also been revealed in neonatally clomipramin-treated rats, a model of depression, in which increased percentage of REM sleep and shortened REM latency onset have also been found [77]. The theta increase in NREM sleep was associated with less restful sleep, probably due to decreased monoaminergic signalling [78]. Based on our results, chronic SSRI administration prevented the elevated theta wave activity during SWS after RD. It suggests that this pharmacotherapy may be efficient in reducing those effects of a stressful SD procedure which also occur in animal models of depression and may indicate a decrease in the quality of sleep.

### Markov-modelling

Another implication of this study was demonstrating the applicability of Markov chains to model hypnograms. We were not the first to use this methodology in sleep research, but we are not aware of any other communication where not proprietary but freely available open-source software was used. Describing and comparing hypnograms with Markov chains greatly facilitated our analysis, because the parameters were easily interpretable in biological context and this statistical methodology seemed to have higher statistical power than the standard approach. However, we came to our positive conclusions using data of a relatively short (2 h) sleep study, fitting time-continuous Markov model to the data of longer time periods would be a more challenging task. The computational time increases more than linearly with the number of observations and finding the breakpoints for the correct segmentation would demand multiple runs.

Another issue is that we used a relatively simple sleep model with only two or three stages while in sleep research, studies typically distinguish many more. In fact, we could not fit more complex models. But none of these limitations are due to Markov modelling methodology itself, but rather they reflect hardware and software problems we faced.

## Conclusions

Chronic escitalopram attenuated the accelerated NREM↔REM transitions during the rebound sleep and the increased rate of awakenings in the first 30 min of the rebound. In addition, the RD-caused elevation in theta power density during SWS stages was also reduced by escitalopram. However, the SSRI did not suppress the REM rebound (increased time spent in REM, decreased REM latency) and did not influence the decelerated transitions to falling asleep in the steady phase (~30-120 min) of sleep. Therefore, normalization in sleep transitions and EEG spectra can be characteristic features of SSRIs and potentially other antidepressants, which also support the validity of the flower pot paradigm to mimic pathological conditions such as anxiety or depression, at least regarding sleep. Finally we showed that time-continuous Markov-modelling is a powerful statistical tool which might allow analyzing hypnograms in finer granularity and with higher statistical power than the standard sleep metrics. Currently the method is not without any limitations but the potential area of application is wide and even now can be a reasonable alternative to the standard approach.
